# Interleukin-8 is superior to CRP for the prediction of severe complications in a prospective cohort of patients undergoing major liver resection

**DOI:** 10.1007/s00423-023-03041-w

**Published:** 2023-09-25

**Authors:** Mathieu Pecqueux, Frederik Brückner, Andreas Bogner, Florian Oehme, Hans‑Michael Hau, Felix von Bechtolsheim, Hanns‑Christoph Held, Franziska Baenke, Marius Distler, Carina Riediger, Jürgen Weitz, Christoph Kahlert

**Affiliations:** 1 Department of Visceral, Thoracic and Vascular Surgery, Faculty of Medicine and University Hospital Carl Gustav Carus, Technische Universität Dresden, Fetscherstrasse 74, 01307 Dresden, Germany; 2 National Center for Tumor Diseases (NCT/UCC), Dresden, Germany: German Cancer Research Center (DKFZ), Heidelberg, Germany; Faculty of Medicine and University Hospital Carl Gustav Carus, Technische Universität Dresden, Dresden, Germany; Helmholtz-Zentrum Dresden - Rossendorf (HZDR), Dresden, Germany

**Keywords:** Liver surgery, Hepatic surgery, Complication, Interleukin-8, IL-8, Perioperative surgery, Inflammation

## Abstract

**Introduction:**

Early detection of severe complications may reduce morbidity and mortality in patients undergoing hepatic resection. Therefore, we prospectively evaluated a panel of inflammatory blood markers for their value in predicting postoperative complications in patients undergoing liver surgery.

**Methods:**

A total of 139 patients undergoing liver resections (45 wedge resections, 49 minor resections, and 45 major resections) were prospectively enrolled between August 2017 and December 2018. Leukocytes, CRP, neutrophil-lymphocyte ratio (NLR), thrombocyte-lymphocyte ratio (TLR), bilirubin, INR, and interleukin-6 and -8 (IL-6 and IL-8) were measured in blood drawn preoperatively and on postoperative days 1, 4, and 7. IL-6 and IL-8 were measured using standardized immunoassays approved for in vitro diagnostic use in Germany. ROC curve analysis was used to determine predictive values for the occurrence of severe postoperative complications (CDC ≥ 3).

**Results:**

For wedge and minor resections, leukocyte counts at day 7 (AUC 0.80 and 0.82, respectively), IL-6 at day 7 (AUC 0.74 and 0.73, respectively), and CRP change (∆CRP) at day 7 (AUC 0.72 and 0.71, respectively) were significant predictors of severe postoperative complications. IL-8 failed in patients undergoing wedge resections, but was a significant predictor of severe complications after minor resections on day 7 (AUC 0.79), had the best predictive value in all patients on days 1, 4, and 7 (AUC 0.72, 0.72, and 0.80, respectively), and was the only marker with a significant predictive value in patients undergoing major liver resections (AUC on day 1: 0.70, day 4: 0.86, and day 7: 0.92). No other marker, especially not CRP, was predictive of severe complications after major liver surgery.

**Conclusion:**

IL-8 is superior to CRP in predicting severe complications in patients undergoing major hepatic resection and should be evaluated as a biomarker for patients undergoing major liver surgery. This is the first paper demonstrating a feasible implementation of IL-8 analysis in a clinical setting.

**Supplementary Information:**

The online version contains supplementary material available at 10.1007/s00423-023-03041-w.

## Introduction

Continued advances in diagnostics and treatments have expanded the indication and extent of liver resections. Currently, it has become an established curative treatment option, particularly for hepatic metastases from colorectal cancer. The success in terms of 5- and 10-year survival rates in patients undergoing curative R0 resections for metastatic colorectal cancer has dramatically increased the frequency and extent of hepatic resections [1]. These extensive resections in increasingly multimorbid patients have resulted in postoperative complication rates of up to 50% [2], which may impact not only quality of life but also overall survival [3].

Early diagnosis of complications could mitigate their negative impact on patients’ overall outcome of patients by reducing morbidity, hospital stay and cost, and improving quality of life and overall survival. Several cellular and acellular blood components belong to the standard repertoire for the detection of postoperative complications, as these markers are subject to significant changes during acute or chronic inflammatory responses.

Serum C-reactive protein (CRP) is synthesized by the liver upon secretion of IL-6 by T cells and macrophages. CRP is a robust and well-described marker in elective colorectal cancer resection [4, 5], but a retrospective study has shown the limited predictive power of CRP in patients undergoing major liver resection [6]. The neutrophil-lymphocyte ratio (NLR) and thrombocyte-lymphocyte ratio (TLR) are primarily liver independent and have shown some predictive power for complications in elective gastric surgery and in intensive care patients [7–9], as well as predictive power for poor survival in several cancers, including colorectal cancer, hepatocellular carcinoma, and hepatopancreaticobiliary malignancies [10–12]. Proinflammatory cytokines, such as IL-6 and IL-8, are predominantly produced at the site of infection and can therefore be considered early response markers of the acute phase response (APR). They are closely associated with inflammation, liver disease, and liver regeneration [13, 14]. IL-6 is secreted by T lymphocytes, endothelial cells, and macrophages. It reaches the liver via the bloodstream and induces APR, namely, the production of CRP in the liver. IL-8 is produced by macrophages, epithelial cells, endothelial cells, and fibroblasts and initiates the recruitment of neutrophils; thus, its release is upstream of the NLR. Both proinflammatory cytokines have shown promising results in critically ill patients and in acute pancreatitis [15, 16], but have not yet sufficiently been described in liver resected patients.

The aim of the present study was to identify predictive markers for the development of severe postoperative complications (Clavien-Dindo ≥ 3) in patients undergoing various degrees of liver resection and to demonstrate the feasibility of IL-8 analysis in a clinical setting.

## Methods

### Patient cohort

The current study is a prospective single-center cohort study evaluating the prognostic value of different serum markers for predicting severe complications (Clavien-Dindo ≥ 3) in patients undergoing liver resection. The results were reported according to the STROCSS criteria [17]. This study was approved by the Ethics Committee on July 20, 2017. A total of 151 patients who underwent hepatic resection for benign or malignant liver disease (*Institution information double blinded*) were prospectively enrolled between August 02, 2017, and December 13, 2018. All patients provided written informed consent prior to enrollment in the study. Postoperatively, 12 out of 151 patients were excluded due to irresectability (*n* = 3), exclusive liver biopsies (*n* = 5), or two-stage liver resections (*n* = 4). The remaining 139 patients were included in the study.

Data completeness was 71% preoperatively, 88% on day 1, 85% for day 4, and 60% on day 7. Missing data can be explained by transition from the ICU to the regular ward or by patients who underwent minor resections and were discharged before postoperative day 7.

### Operation

All patients received a preoperative computed tomography (CT) scan of the abdomen to assess the extent of the resection and a CT scan of the chest to exclude pulmonary metastases in patients with malignant disease. Functional liver remnant (FLR) was assessed prior to major liver resection. Patients, whose remaining liver volume was estimated to be less than 30% or less than 40–50% in patients with pre-existing cirrhosis, cholestasis, fibrosis, or prolonged neoadjuvant chemotherapy, received preoperative portal vein embolization to increase the volume of the FLR. Cholestasis was treated preoperatively by endoscopic retrograde cholangiopancreaticography or bercutaneous transhepatic cholangiography. Patients with no increase in FLR after portal vein embolization or with a very low initial FLR were considered for ALPPS (Associating Liver Partition and Portal Vein Ligation for Staged Hepatectomy). Preoperative, perioperative, and postoperative care was standardized.

Resections were performed by crush-clamp in combined with diathermy or LigaSure (laparoscopic). The Pringle maneuver was used only in cases of bleeding. Liver resection was performed at the surgeon’s discretion under low central venous pressure (< 5 mmHg).

### Evaluation of perioperative morbidity and mortality

Perioperative morbidity and mortality was defined as any complication or death occurring up to 90 days after surgery. Complications were graded according to the Clavien-Dindo Classification (CDC) [18] and dichotomized into minor (CDC 0–2) and severe (CDC 3–5) complications.

### Serum marker analysis and predictive value

Serum samples were analyzed preoperatively and on day 1, day 4, and day 7 after resection according to the protocol. The serum panel included standard markers such as leukocytes (GPt/L), platelet count (GPt/L), CRP (mg/L), bilirubin (μmol/L), and international normalized ratio (INR) as well as a differential blood count to calculate the NLR and TLR. In addition, IL-6 and IL-8 were determined as part of the standardized laboratory diagnostics. Our clinical laboratory uses an Immulite 1000 (Siemens) device, which is approved for in vitro diagnostics in Germany and uses standardized assays to assess and validate serum IL-6 and IL-8 levels. The cost of the test is estimated at 10–15 Euros per test, making it possible to include it in routine diagnostics for specific cases, such as major liver resection.

The dynamic values (∆CRP, ∆IL-6, and ∆IL-8) were calculated by subtracting the value on day 1 from the value on day 4 (∆day4) or by subtracting the value on day 4 from the value on day 7 (∆day7).

The predictive value was calculated for all patients and separately for the subgroups of patients who underwent wedge liver resection (wedge resections with minimal loss of normal liver tissue), minor liver resection (segment/bisegment resections with moderate loss of normal liver tissue), and major liver resection (≥3 segments with severe loss of normal liver tissue). Receiver operating characteristic (ROC) curves were plotted and the area under the curve (AUC) was calculated. AUCs ≥ 0.7 were defined as acceptable, AUCs ≥ 0.8 as excellent, and AUCs ≥ 0.9 as exceptional biomarkers [19]. The optimal cutoff was determined using the Youden index, and sensitivity, specificity, and *p*-value (chi-squared) were subsequently calculated.

### Statistics

Statistical analysis was performed with SPSS (IBM, version 26). ROC curves in combination with the Youden index (sensitivity + specificity − 1) were used to define cutoff values. *p*-values were calculated using the *t*-test comparing the values in the group with and those in the group without complications, and using the chi-squared test using the optimal cutoff value (Youden test) to evaluate the predictive value, *p*-values < 0.05 were considered statistically significant. Patients with severe complications before day 1, 4, or 7 were censored for the calculation of AUC, sensitivity, specificity, and *p*-values (chi-squared and *t*-test) on day 1, 4, or 7, respectively.

## Results

A total of 139 patients undergoing hepatic resection were prospectively enrolled in the study. The group consisted of 56 female (40%) and 83 male (60%) patients with a mean age of 61 years (27–84 years). Only 4% of patients had an ASA (American Society of Anesthesiologists) score of 1, 30% had an ASA score of 2, and 67% had an ASA score of 3. Almost all patients underwent liver resection for malignant disease (*n* = 128; 92%), with most resections performed for colorectal liver metastases (*n* = 78), followed by cholangiocarcinoma (*n* = 21), HCC (*n* = 17), and other metastases (*n* = 12). Of the 139 patients, 45 patients (32%) underwent wedge resections, 49 (35%) underwent minor resections, and 45 (32%) underwent major resections (≥ 3 liver segments). Further characteristics of the cohort in terms of clinical data, complications (CDC), and the operations performed are shown in Table 1 and Supplementary Table 1.

**Table 1:** Clinicopathological characteristics of 138 patients undergoing liver resection (values in parentheses are percentages unless otherwise stated)

	Wedge resections (*n* = 45)	Minor resections ≤ 2 segments (*n* = 49)	Major resections (*n* = 45)	All (*n* = 139)
Age (mean + range)	60 (35–82)	62 (28–84)	63 (27–81)	61 (27–84)
Sex f/m (f%/m%)	11/34 (25%/75%)	25/24 (51%/49%)	20/25 (44%/56%)	56/83 (40%/60%)
ASA I	2 (4%)	2 (4%)	1 (2%)	5 (4%)
II	15 (33%)	13 (27%)	13 (29%)	41 (30%)
III	28 (62%)	34 (69%)	31 (69%)	93 (67%)
Neoadj. chemo	15 (34%)	13 (27%)	17 (38%)	45 (32%)
Benign	5 (11%)	3 (6%)	3 (7%)	11 (8%)
Malignant	39 (89%)	46 (94%)	42 (93%)	128 (92%)
CRC met	22 (49%)	30 (61%)	26 (58%)	78 (56%)
HCC	5 (11%)	9 (18%)	3 (7%)	17 (12%)
CCC	5 (11%)	4 (8%)	12 (27%)	21 (15%)
Other met	8 (18%)	3 (6%)	1 (2%)	12 (9%)
Prev. liver res.	19 (42%)	16 (33%)	27 (60%)	62 (45%)
Open surgery	36 (80%)	32 (65%)	43 (96%)	111 (80%)
Laparoscopic surgery	9 (20%)	17 (35%)	2 (4%)	28 (20%)
No complication	31 (69%)	32 (65%)	12 (27%)	75 (54%)
**≥ **1 complication	14 (31%)	17 (35%)	33 (73%)	64 (46%)
**≥ **1 major compl.	8 (18%)	9 (18%)	21 (47%)	38 (27%)
Clavien-Dindo				
CD I	2 (4%)	6 (12%)	4 (9%)	12 (9%)
CD II	5 (11%)	6 (12%)	11 (24%)	22 (16%)
CD IIIa	9 (20%)	8 (16%)	9 (20%)	26 (19%)
CD IIIb	2 (4%)	4 (8%)	9 (20%)	15 (11%)
CD IV	0	0	2 (4%)	2 (1%)
CD V	0	0	5 (11%)	5 (4%)
Days ICU mean (min–max)	1 (0–10)	1 (0–15)	8 (0–82)	3 (0–82)
Days hospitalized (min–max)	11 (3–36)	11 (5–37)	20 (6–102)	14 (3–102)

*ASA indicates the American Society of Anesthesiologists score; CRC indicates colorectal cancer; HCC indicates hepatocellular cancer; CCC indicates cholangiocellular cancer; ICU indicates intensive care unit stay; Prev. liver res. indicates previous liver resections; Discrepancies between number of complications can result from patients having more than one complication

A total of 75 complications occurred in 64 out of 139 patients (46%). Minor complications (CDC I–II) were observed in 26 patients (19%), while 33 patients (24%) had at least one severe complication (CDC III–IV), and 5 patients (4%) died during hospitalization. Major liver resections were associated with more complications and longer intensive care unit (ICU) stay than wedge or minor resections (Tables 1 and 2).

**Table 2:** Surgery related and medical morbidity

	Wedge resections (*n* = 44)	Minor resections ≤ 2 segments (*n* = 49)	Major resections (*n* = 45)	All resections (*n* = 138)
Perioperative morbidity				
Wound infection	2 (5%)	5 (10%)	6 (13%)	13 (9%)
Biliary leakage	2 (5%)	2 (4%)	5 (11%)	9 (6%)
Abscess	1 (2%)	2 (4%)	4 (9%)	7 (5%)
Bilioma	2 (5%)	1 (2%)	3 (7%)	6 (4%)
Fluid retention	1 (2%)	2 (4%)	3 (7%)	6 (4%)
Intestinal atony	2 (5%)	0	2 (4%)	3 (2%)
Hemorrage	1 (2%)	0	2 (4%)	3 (2%)
Hematoma	2 (5%)	1 (2%)	1 (2%)	4 (3%)
Liver failure	0	0	4 (9%)	4 (3%)
Intestinal ischemia	0	0	3 (7%)	3 (2%)
Abdominal wall dehiscence	0	0	2 (4%)	2 (1%)
Portal vein thrombosis	0	0	1 (2%)	1 (1%)
Sepsis	0	1 (2%)	0	1 (1%)
Reduced liver perfusion	0	0	1 (2%)	1 (1%)
Small bowel perforation	0	1 (2%)	0	1 (1%)
Pancreatic fistula	0	1 (2%)	0	1 (1%)
Medical complications				
Pulmonary artery embolism	0	2 (4%)	2 (4%)	4 (3%)
Pneumonia	0	0	2 (4%)	2 (1%)
Cardiocirculatory compl.	1 (2%)	0	2 (4%)	3 (2%)
Pleural effusion	1 (2%)	2 (4%)	1 (2%)	4 (3%)
Allergic reaction	1 (2%)	0	0	1 (1%)
Cystitis	0	0	1 (2%)	1 (1%)

One-quarter of the complications occurred before day 4 (*n* = 21; 25%), 14 (17%) occurred between days 4 and 6, 34 (41%) between days 7 and 14, 9 (11%) between days 15 and 30, and 5 (6%) between days 30 and 90. Fifteen patients (11%) had major complications by day 6, 2 after wedge resections, 4 after segmental resections, and 9 after major liver surgery. Of these, 11 patients (8%) had major complications before day 4. Complications before day 4 consisted mostly of early biliary leaks, sometimes leading to vascular erosion with hemorrhage, which were detected by drainage fluid inspection, as well as some medical complications such as pulmonary embolism or one case of a brief episode of postoperative asystole of unclear origin that requiring brief CPR and the administration of atropine (Supplementary Fig. 1).

### The inflammatory marker leucocytes and CRP can predict complications in patients undergoing minor liver resections

**Fig. 1:**
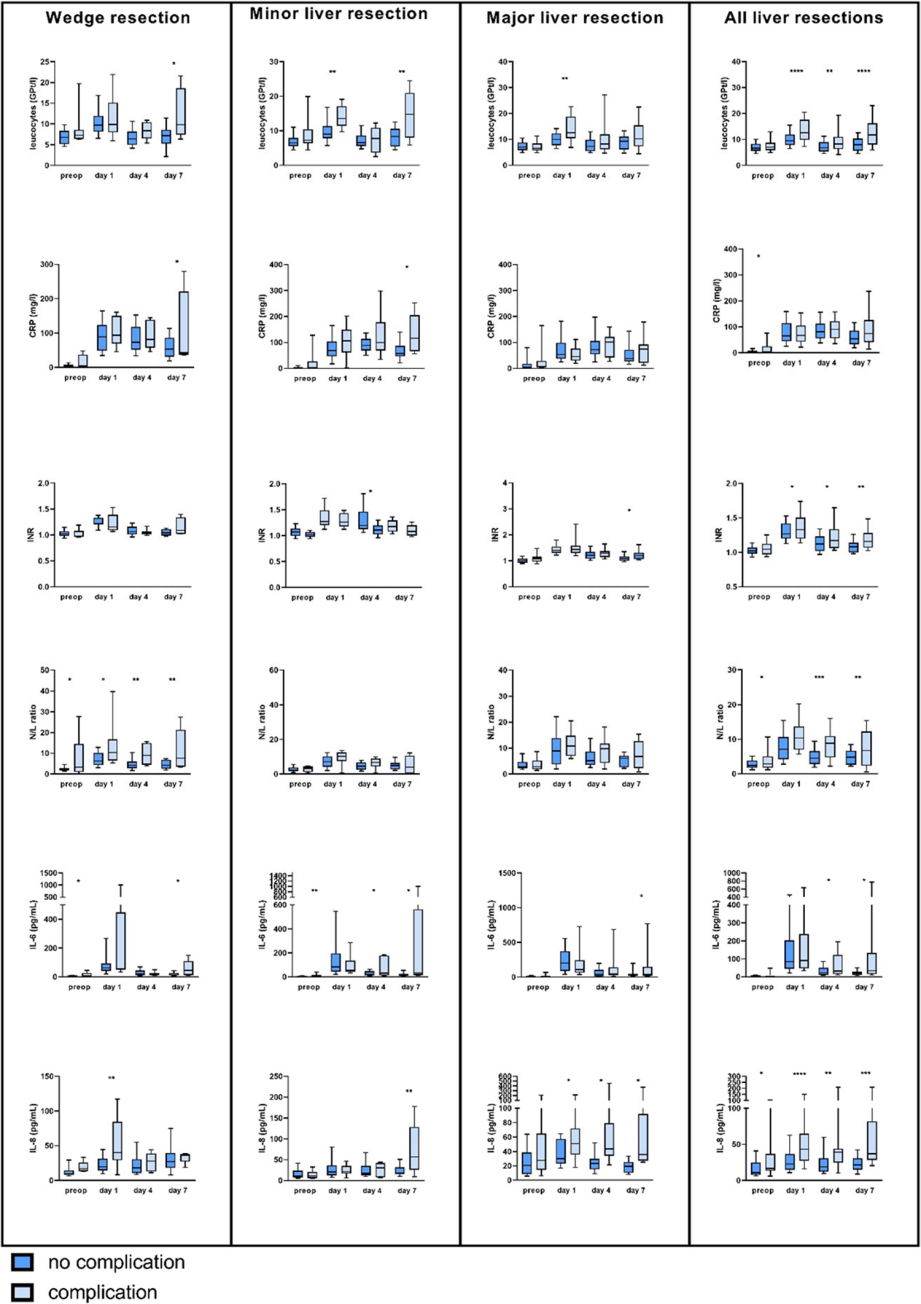
Serum markers in patients with (light blue) or without (dark blue) severe complication after undergoing liver surgery (wedge resection, minor resection, or major resection) preoperatively and at days 1, 4, and 7 after surgery. Values were compared using the *t*-test, values ≤ 0.05 were considered significant

Leukocytes showed acceptable sensitivity and specificity for predicting postoperative complications ≥ CDC III in the whole cohort on day 1 and day 7 (day 1: AUC 0.71, sensitivity 68%, specificity 71%, *p* = 0.001 and day 7: AUC 0.73, sensitivity 50%, specificity 93%, *p* = 0.001). In the subgroup analysis, the AUC showed discriminative values in patients undergoing wedge resection on day 4 (AUC 0.71, sensitivity 57%, specificity 81%, *p* = 0.035) and at day 7 (AUC 0.80, sensitivity 80%, specificity 74%, *p* = 0.027), in patients undergoing minor liver resection on day 1 (AUC 0.83, sensitivity 89%, specificity 73%, *p* = 0.001) and day 7 (AUC 0.82, sensitivity 67%, specificity 95%, *p* = 0.001) and in patients undergoing major liver resection on day 1 (AUC 0.70, sensitivity 40%, specificity 95%, *p* = 0.004) (Table 3/Fig. 1).

**Table 3:** Correlation of serum markers with the occurrence of severe postoperative complications (Clavien-Dindo III–V)

		Preop					Day 1					Day 4					Day 7				
		AUC	Cutoff	Sens	Spec	*p*-value	AUC	Cutoff	Sens	Spec	*p*-value	AUC	Cutoff	Sens	Spec	*p*-value	AUC	Cutoff	Sens	Spec	*p*-value
Wedge resections	Leuc	0.62	6.18	100%	47%	0.013	0.56	9.59	75%	50%	0.199	0.71	8.37	57%	81%	0.035	0.80	8.46	80%	74%	0.027
	NLR	0.58	4.67	50%	96%	0.001	0.74	5.34	100%	43%	0.031	0.80	7.98	67%	89%	0.002	0.76	7.42	60%	94%	0.004
	TLR	0.57	236.84	50%	89%	0.022	0.64	160.00	75%	54%	0.135	0.58	152.87	100%	32%	0.105	0.40	231.18	40%	67%	0.782
	CRP	0.64	2.15	88%	44%	0.093	0.58	66.20	86%	43%	0.155	0.57	80.45	71%	55%	0.209	0.51	35.55	100%	32%	0.246
	IL-6	0.74	3.90	67%	77%	0.038	0.56	495.00	25%	97%	0.032	0.47	10.60	100%	21%	0.187	0.74	44.65	60%	94%	0.004
	IL-8	0.81	12.60	100%	69%	0.004	0.77	29.15	86%	75%	0.002	0.63	24.60	67%	64%	0.162	0.61	32.35	80%	65%	0.078
	Bili	0.68	13.40	63%	89%	0.001	0.64	14.25	75%	53%	0.155	0.70	9.95	57%	84%	0.021	0.72	6.00	100%	56%	0.044
	INR	0.40	0.95	100%	11%	0.315	0.41	1.42	25%	97%	0.036	0.45	0.99	100%	19%	0.205	0.69	1.14	50%	94%	0.019
	∆CRP											0.38	−81.35	100%	17%	0.281	0.72	−49.90	100%	47%	0.081
	∆IL-6											0.49	−38.60	71%	65%	0.080	0.90	3.95	80%	94%	0.001
	∆IL-8											0.27	4.85	17%	84%	0.968	0.41	0.20	80%	40%	0.417
Minor resections	Leuc	0.66	6.99	67%	68%	0.057	0.83	10.98	89%	73%	0.001	0.53	8.37	50%	76%	0.193	0.82	12.93	67%	95%	0.001
	NLR	0.57	2.42	75%	57%	0.233	0.69	9.36	75%	69%	0.023	0.69	5.70	83%	63%	0.035	0.45	8.89	40%	89%	0.116
	TLR	0.53	119.54	75%	47%	0.412	0.49	116.39	75%	40%	0.428	0.41	121.68	83%	34%	0.391	0.43	240.88	40%	79%	0.384
	CRP	0.62	1.55	89%	41%	0.091	0.64	97.50	67%	72%	0.030	0.59	113.30	50%	76%	0.193	0.78	56.55	100%	50%	0.027
	IL-6	0.75	4.45	67%	82%	0.014	0.43	33.00	100%	17%	0.244	0.65	142.00	33%	100%	0.001	0.73	31.85	60%	89%	0.019
	IL-8	0.44	14.15	50%	66%	0.474	0.53	16.85	86%	42%	0.170	0.53	24.25	67%	68%	0.109	0.79	40.35	80%	89%	0.002
	Bili	0.61	8.05	78%	58%	0.056	0.54	8.80	100%	25%	0.093	0.71	8.85	67%	80%	0.017	0.52	6.60	67%	61%	0.237
	INR	0.64	1.04	67%	60%	0.146	0.56	1.46	33%	85%	0.199	0.75	1.15	83%	67%	0.021	0.71	1.14	67%	74%	0.073
	∆CRP											0.54	31.95	67%	56%	0.313	0.71	−29.45	67%	85%	0.012
	∆IL-6											0.85	−21.30	80%	81%	0.005	0.50	−3.50	40%	88%	0.172
	∆IL-8											0.69	0.25	80%	63%	0.074	0.86	0.3	100%	63%	0.015
Major resections	Leuc	0.43	11.69	10%	100%	0.122	0.70	14.84	40%	96%	0.004	0.58	10.93	40%	83%	0.122	0.64	13.43	46%	94%	0.008
	NLR	0.44	5.30	22%	88%	0.412	0.58	4.62	100%	32%	0.006	0.60	9.11	57%	81%	0.020	0.55	9.91	38%	100%	0.006
	TLR	0.49	277.32	28%	100%	0.019	0.55	179.30	60%	64%	0.126	0.55	252.61	62%	71%	0.058	0.51	231.21	46%	75%	0.233
	CRP	0.57	1.30	95%	29%	0.038	0.40	41.55	65%	39%	0.780	0.56	94.25	60%	74%	0.037	0.56	72.75	54%	78%	0.069
	IL-6	0.42	38.65	14%	94%	0.401	0.39	984	5%	100%	0.323	0.58	15.80	92%	29%	0.143	0.58	28.10	67%	56%	0.229
	IL-8	0.63	12.75	86%	47%	0.052	0.70	34.80	80%	65%	0.004	0.86	34.70	79%	91%	0.000	0.92	25.70	92%	81%	0.000
	Bili	0.63	10.90	57%	70%	0.074	0.66	25.5	70%	65%	0.021	0.56	16,00	63%	61%	0.151	0.62	14.25	62%	78%	0.027
	INR	0.61	1.11	48%	83%	0.025	0.53	1.57	26%	87%	0.276	0.56	1.10	88%	26%	0.301	0.69	1.20	54%	83%	0.029
	∆CRP											0.62	18.20	67%	61%	0.097	0.65	−30.70	75%	56%	0.098
	∆IL-6											0.62	−72.20	62%	68%	0.208	0.53	−16.40	78%	47%	0.231
	∆IL-8											0.45	6.15	21%	95%	0.143	0.36	−31.4	90%	13%	0.846
All patients	Leuc	0.56	5.71	87%	30%	0.045	0.71	11.07	68%	71%	0.001	0.63	8.08	57%	70%	0.009	0.73	13.09	50%	93%	0.001
	NLR	0.57	3.9	43%	76%	0.056	0.69	6.53	89%	48%	0.001	0.71	8.65	54%	88%	0.001	0.59	9.11	39%	96%	0.001
	TLR	0.59	279	29%	96%	0.001	0.58	175.2	58%	60%	0.064	0.52	232	44%	68%	0.258	0.45	231.18	43%	70%	0.240
	CRP	0.64	1.35	95%	29%	0.003	0.47	79.4	44%	59%	0.706	0.56	80.65	71%	49%	0.118	0.60	66.95	57%	67%	0.049
	IL-6	0.61	3.55	58%	68%	0.018	0.52	33.00	97%	16%	0.046	0.61	120.50	27%	95%	0.001	0.69	29.25	64%	75%	0.002
	IL-8	0.65	13.75	76%	63%	0.001	0.72	32.45	71%	72%	0.001	0.72	24.35	81%	65%	0.001	0.80	26.60	82%	67%	0.001
	Bili	0.65	13.15	47%	82%	0.001	0.68	24.7	46%	82%	0.001	0.69	8.35	79%	56%	0.001	0.66	14.25	48%	87%	0.001
	INR	0.58	1.12	37%	87%	0.001	0.58	1.44	42%	77%	0.036	0.64	1.15	62%	62%	0.026	0.72	1.15	61%	76%	0.002
	∆CRP											0.56	0.75	74%	43%	0.104	0.70	−30.7	73%	63%	0.005
	∆IL-6											0.58	−37.60	56%	72%	0.010	0.61	3.95	37%	89%	0.012
	∆IL-8											0.43	4.45	28%	84%	0.166	0.51	0.20	60%	53%	0.323

AUC indicates area under the curve; *Leuc*, leucocytes; *NLR*, neutrophil-lymphocyte ratio; *TLR*, thrombocyte-lymphocyte ratio; *CRP*, C-reactive protein; *IL-6*, interleukin-6; *IL-8*, interleukin-8; *Bili*, bilirubin; *INR*, international normalized ratio

**Fig. 2:**
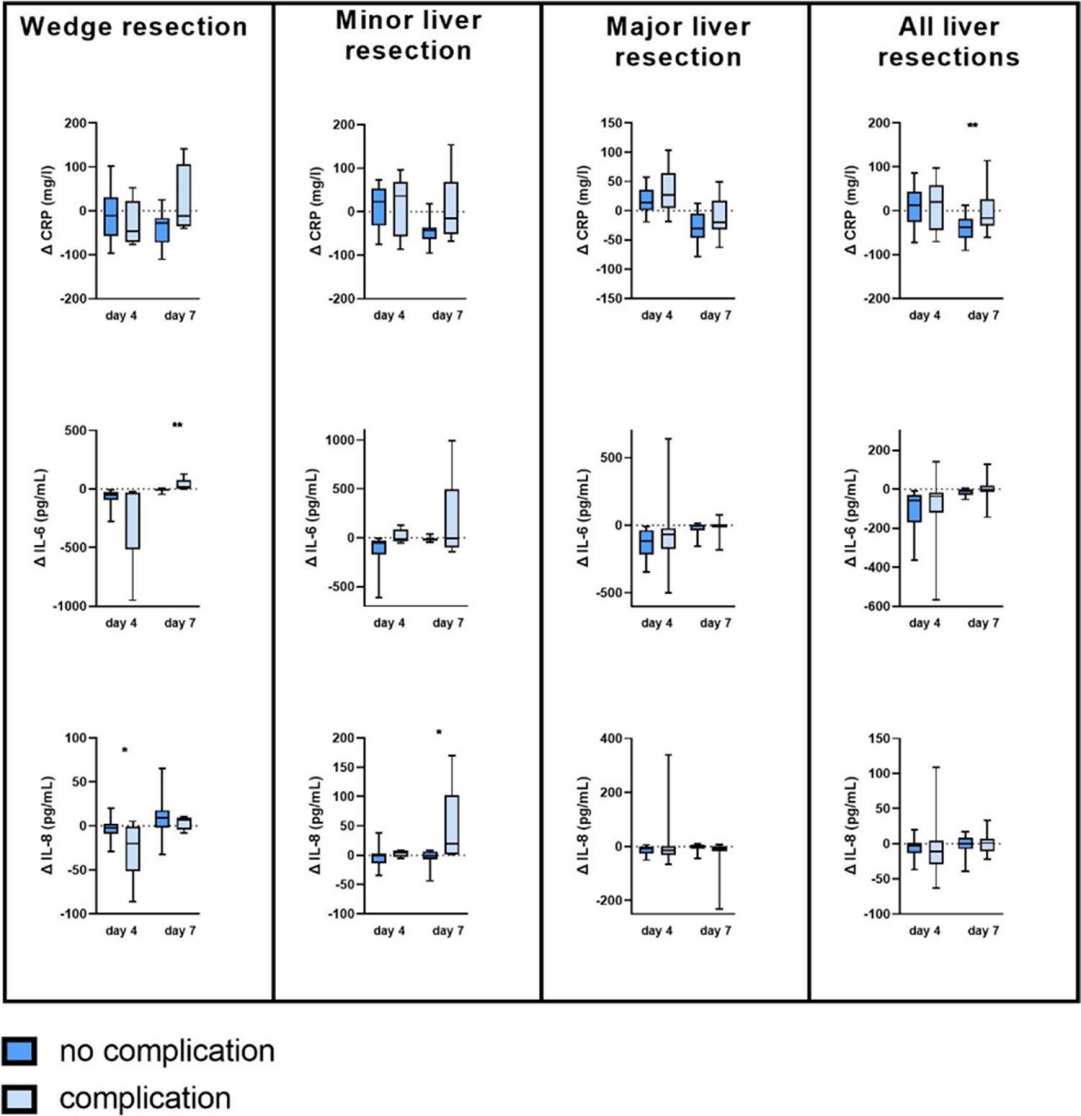
Changes in CRP, interleukin-6 (IL-6), and interleukin-8 (IL-8) in patients with (light blue) or without (dark blue) severe complication after undergoing liver surgery (wedge resection, minor resection, or major resection). The values were calculated using the following formula: Δ day 4 = value day 4 − value day 1; Δ day 7 = value day 7 − value day 4. *p*-values were calculated using the *t*-test, values ≤ 0.05 were considered significant

**Fig. 3:**
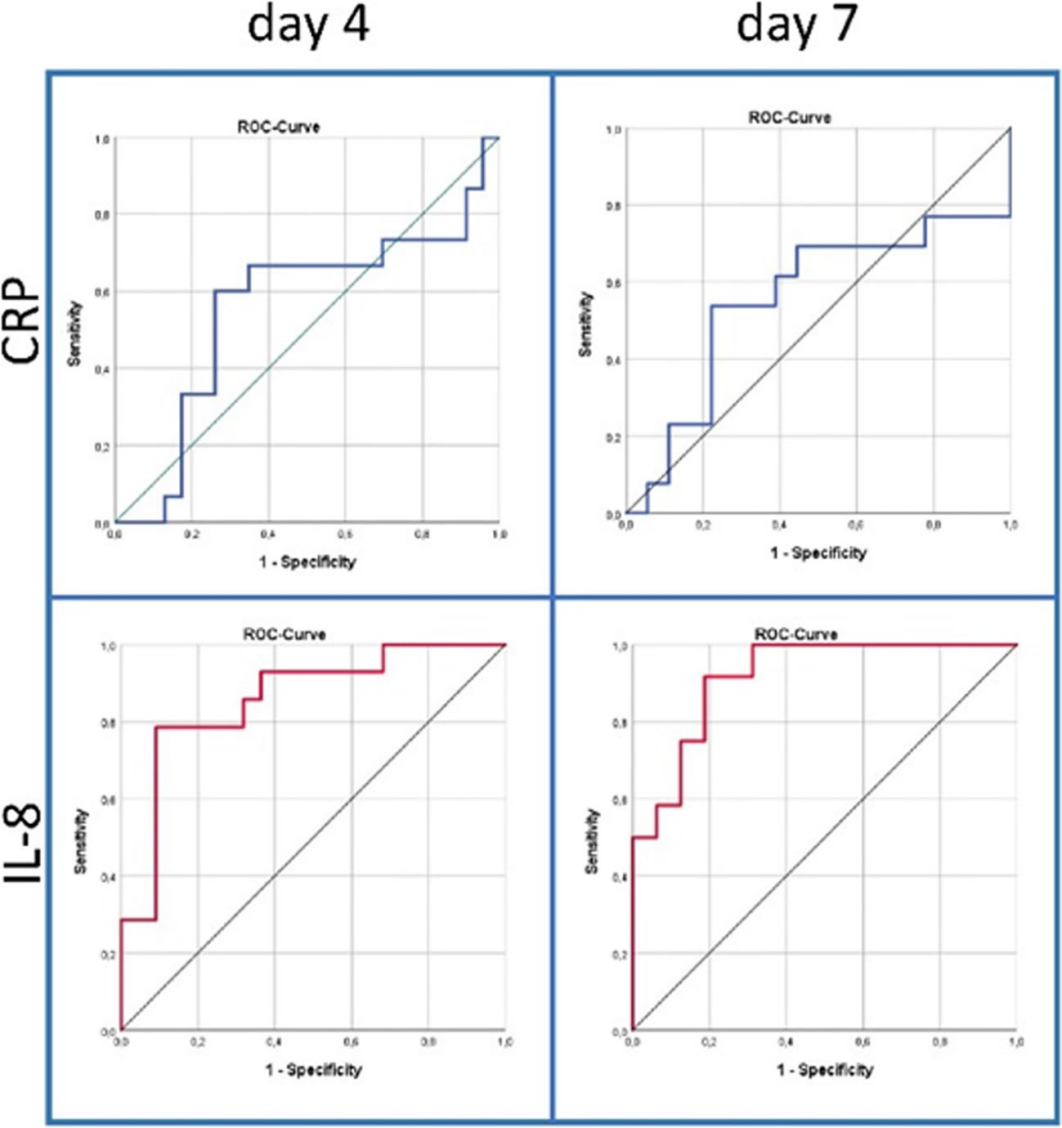
ROC curves of IL-8 and CRP at day 4 and day 7 after surgery in patients undergoing major liver resections

While CRP was a weak surrogate marker for predicting severe complications (≥ CDC III) in all patients, subgroup analysis revealed an acceptable predictive value for patients undergoing minor liver resection on postoperative day 7 (AUC = 0.78, sensitivity 100%, specificity 50%, *p* = 0.027). In particular, dynamic CRP levels (∆CRP) at day 7 showed acceptable discriminative values in all patients (AUC 0.70, sensitivity 73%, specificity 63%, *p* = 0.005) as well as in the subgroups of patients undergoing wedge resection (AUC 0.72, sensitivity 100%, specificity 47%, *p* = 0.081) and minor resection (AUC 0.71, sensitivity 67%, specificity 85%, *p* = 0.027). In the subgroup of patients who underwent major liver resection, CRP showed a very poor and non-significant correlation with severe complications on any postoperative day (Table 3; Figs. 1, 2 and 3).

### The liver function markers bilirubin and INR can predict complications in wedge resection and minor resection

Elevated bilirubin was a significant surrogate marker for complications only in patients who underwent wedge liver resection at 4 (AUC 0.70, sensitivity: 57%, specificity: 84%, *p* = 0.021) and 7 postoperative days (AUC 0.72, sensitivity: 100%, specificity: 56%, *p* = 0.044) and in patients who underwent minor liver resection at 4 postoperative days (AUC 0.71, sensitivity: 67%, specificity: 80%, *p* = 0.017). However, there was no significant association between elevated bilirubin and the occurrence of severe complications in patients with major resection and in all patients (Table 3, Fig. 1). The INR showed an acceptable AUC to discriminate patients with and without severe complications (≥ CDC III) on day 7 in all patients (AUC 0.72, sensitivity: 61%, specificity: 76%, *p* = 0.0002) and in patients undergoing minor resection on day 4 (AUC = 0.75, sensitivity: 83%, specificity: 67%, *p* = 0.021) and day 7 (AUC 0.71, sensitivity: 67%, specificity: 74%, *p* = 0.073). INR did not achieve predictive UACs in patients undergoing wedge or major liver resections (Table 3, Fig. 1).

### NLR is a surrogate marker for complications only in patients with wedge resection

The NLR in the subgroup of patients with wedge resection revealed predictive values on day 4 and at day 7 (day 4: AUC 0.80, sensitivity: 67%, specificity: 89%, *p* = 0.002; day 7: AUC 0.76, sensitivity: 60%, specificity: 94%, *p* = 0.004), with an acceptable predictive value on day 4 in all samples (AUC 0.71, sensitivity: 54%, specificity: 88%, *p* = 0.001).

In contrast, TLR did not show an acceptable predictive value (Table 3, Fig. 1).

### The predictive value of the proinflammatory cytokines IL-6 and IL-8 in wedge, minor, and major liver resections

IL-6 was useful in predicting patients with and without severe complications after wedge resection or minor resection preoperatively (wedge resection: AUC 0.74, sensitivity: 67%, specificity: 77%, *p* = 0.038; minor resection AUC 0.75, sensitivity: 67%, specificity: 82%, *p* = 0.014) and at postoperative day 7 (wedge resection: AUC 0.74, sensitivity: 60%, specificity: 94%, *p* = 0.004 and minor resection: AUC 0.73, sensitivity: 60%, specificity: 89%, *p* = 0.019). However, IL-6 showed a poor correlation with severe complications in patients undergoing major liver resection as well as in all resected patients (Table 3, Fig. 1).

IL-8 showed the best diagnostic value for predicting complications in all patients on postoperative day 1 (AUC 0.72, sensitivity: 71%, specificity: 72%, *p* = 0.001), day 4 (AUC 0.72, sensitivity: 81%, specificity: 65%, *p* = 0.001), and day 7 (AUC 0.80, sensitivity: 82%, specificity: 67%, *p* = 0.001). In the subgroup analyses, IL-8 showed good results in patients who underwent wedge resection preoperatively (AUC 0.81, sensitivity: 100%, specificity: 69%, *p* = 0.004) and on postoperative day 1 (AUC 0.77, sensitivity: 86%, specificity: 75%, *p* = 0.002) and in patients who underwent minor resection on postoperative day 7 (AUC 0.79, sensitivity: 80%, specificity: 89%, *p* = 0.002). Most importantly, IL-8 was the only serum marker that showed an excellent predictive potential in distinguishing patients with severe complications after receiving major liver resection on day 4 (AUC 0.86, sensitivity: 79%, specificity: 91%, *p* = 0.000) and day 7 (AUC 0.92, sensitivity: 92%, specificity: 81%, *p* = 0.000) as well as an acceptable predictive value on day 1 (AUC 0.70, sensitivity: 80%, specificity: 65%, *p* = 0.004) (Table 3, Fig. 1).

Dynamic IL-6 and IL-8 levels (∆IL-6 and ∆IL-8) showed no consistent predictive value (Table 3, Fig. 2).

Thus, IL-8 was the only serum marker that showed a significant predictive value for postoperative complications in patients undergoing major liver resection. In particular, not only conventional markers such as CRP but also newer markers such as N/L and TLR ratio could not reach an acceptable predictive value in these patients (Figs. 1, 2, and 3). This discrepancy is mainly due to the lack of CRP elevation in patients after major liver resection due to loss of liver tissue (Fig. 1).

## Strengths and limitations

Our study is the first to prospectively evaluate a wide range of serum markers in patients undergoing liver resection. The large number of patients included (*n* = 139), as well as the wide spectrum ranging from wedge resections to minor liver resections and major liver resections, provides a representative picture of the clinical reality in a maximum care hospital. The subgroup analyses provide further insight into the clinical issue of detecting major complications in patients undergoing major liver resection. A weakness of the study is the relatively small sample size in the subgroups. In particular, the subgroups of patients who underwent wedge resections and minor resections have fewer major complications (18%), which increases the risk of statistical variance in these groups. Furthermore, the novel serum markers IL-6 and IL-8 are not part of the standard repertoire of every hospital. However, the total number of resected patients is very large (*n* = 139), and the subgroup of patients who underwent major liver resection represents a high-risk cohort with a high complication rate (46% severe complications), making the results reliable. In addition, major hepatic resections are technically complex procedures that require a significant amount of resources and should be reserved for specialized centers. We do not expect any problems with the implementation of IL-8 into their repertoire should these results be confirmed in further studies.

## Discussion

Our prospective study provides a comprehensive summary of the predictive values of a panel of widely used and novel serum markers for the detection of clinically relevant complications in patients undergoing liver surgery. After comparing the predictive values of all markers, leukocytes, CRP, IL-6, and IL-8 showed the most consistent results, with acceptable predictive values (AUC ≥ 0.7) and significant differences (*p* ≤ 0.05) in more than one of our defined subgroups. As commonly used in daily clinical practice, CRP works best when its dynamic course is taken into account (∆CRP). Then, CRP is a robust predictive marker of postoperative outcome for patients undergoing wedge or minor liver resection. However, in patients undergoing major liver surgery, the predictive correlation with postoperative complications is rather poor. These findings are consistent with previous publications of retrospective data [6, 20] and can be explained by the synthesis and release of CRP by the liver, which limits the response after extensive liver tissue resection. In contrast, proinflammatory cytokines, particularly IL-8, showed a stronger correlation with the occurrence of postoperative complications, especially in major liver surgery. IL-6 and IL-8 are both released at the site of inflammation, which should make them independent of liver function and therefore of the extent of liver resection [21, 22]. Nevertheless, IL-6 could be used to predict postoperative complications with relative accuracy only in the subgroup with wedge and minor resections, similar to CRP. This could be explained by the fact that Kupffer cells (resident macrophages of the liver) are an important source of IL-6 [23]. The residual liver tissue after major liver resection may not be sufficient to synthesize IL-6, thus limiting the IL-6 response after extensive liver tissue resection, similar to the CRP response.

In contrast, the release of IL-8 is more independent of functional liver tissue. In addition to macrophages and Kupffer cells, fibroblasts and endothelial cells at the site of infection also secrete IL-8 [22, 24, 25]. The release of IL-8 leads to an increased recruitment of neutrophils, which is less affected by liver function and its residual volume. This may contribute to the fact that IL-8, in particular, is equivalent to CRP after minor surgery and far superior to CRP as a predictor of postoperative severe complications after major liver surgery. The weaker performance in wedge and minor resections is unclear, but might be partly explained by a lower number of major complications in these groups (wedge resections *n* = 8, minor resections *n* = 9), making these groups prone to higher statistical variability. This could also explain why IL-8 performs much better in patients undergoing major liver surgery, as well as in all patients. However, this needs to be confirmed in future studies.

This also provided the rationale to additionally investigate the NLR and TLR as surrogate markers for postoperative complications after liver surgery. Despite being fundamentally liver-independent, thrombocytopenia is a common finding after liver resection [26] and in liver disease [27]. Similarly, neutrophil function is severely impaired in patients with chronic liver dysfunction [28]. Therefore, it was reasonable to hypothesize that NLR or TLR could be a robust surrogate marker for liver dysfunction as well as for posthepatectomy-associated complications. However, in our prospective cohort, we identified NLR solely as a diagnostic marker for postoperative complications in patients undergoing wedge resection, whereas TLR showed no predictive value. Moreover, our investigation revealed that NLR and TLR exhibited only marginal predictive capabilities as markers for postoperative complications in major hepatic surgery. These findings are in line with previous scientific studies, such as the research conducted by McCluney et al., which also reported limited discriminative capacity of NLR in the context of postoperative complications in hepatic surgery [20].

Furthermore, our study findings revealed the occurrence of significant complications in 15 patients (11%) within the initial 6 days following the procedure. Notably, 10 patients (7%) experienced such complications before the fourth postoperative day. The early complications primarily consisted of early biliary leaks which were detected prior to the manifestation of a substantial infectious component, or postoperative hemorrhage, both readily detectable through drainage fluid inspection. Additionally, noninfectious medical complications, such as pulmonary embolism or temporary postoperative asystole of uncertain origin, posed diagnostic challenges when utilizing infectious serum markers.

The apparent limitations of IL-8 as a predictive marker before the fourth day after surgery can be attributed to a combination of factors. Firstly, our analysis considered the aforementioned noninfectious complications, which constituted a significant portion of early adverse events. Secondly, the vast majority (92%) of the patients underwent liver resection for malignant indications, a well-known factor associated with elevated IL-8 levels [29, 30]. Notably, patients with larger tumors undergoing major liver resection exhibited elevated preoperative IL-8 values, which further increased after resection and subsequently normalized around day four in patients without postoperative complications (Fig. 1). Moreover, the act of liver resection itself led to increased postoperative IL-8 values, which normalized by the fourth day. Considering these factors, selecting the fourth day post-surgery appears to strike a reasonable balance between sensitivity and specificity for analyzing IL-8. This is corroborated by the observation of good prognostic differentiation on the fourth day in patients who underwent major liver resection. However, the reasons for IL-8’s diminished prognostic performance in patients undergoing minor liver surgery remain elusive, and potential explanations may be linked to the lower incidence of major complications within this subgroup. Additionally, it is plausible that IL-8’s involvement in cancer metabolism, inflammation, and progression is more pronounced in larger cancers, contributing to the observed differences in prognostic efficacy. These observations may also shed light on the association between preoperative IL-8 levels and the occurrence of major postoperative complications. The presence of an inflamed microenvironment in patients with malignant liver lesions might influence the development of postoperative complications. However, due to the heterogeneity of malignant tumors included in this study, a more comprehensive analysis is not currently feasible. Nevertheless, these results are highly intriguing and warrant confirmation in further studies.

In summary, our study demonstrates that established markers commonly used for clinical monitoring after surgery fail to predict severe complications following major liver surgery. Our data suggest that leukocyte count and dynamic changes in CRP remain reliable markers in patients undergoing wedge or minor liver surgery, but are not suitable for predicting postoperative complications after major liver surgery. Additionally, it should be noted that inflammation markers specific to the liver, such as CRP, are influenced by the remaining liver volume, particularly after major liver resection.

While IL-8 is not a routine marker that is easily measured in every laboratory, our study showcases the feasibility of routine IL-8 assessment in serum using standardized testing equipment, which can easily be reproduced. Although the cost of determining IL-8 in the serum is significant (approximately 10–15 Euros per test), it may be worthwhile to consider its assessment in specific scenarios, such as major liver surgery.

To effectively predict and diagnose complications in these patients during the early postoperative stage, liver-independent markers like IL-8 should be validated in future trials with the aim of incorporating them into the standard repertoire of blood tests in the future.

### Supplementary Information

Below is the link to the electronic supplementary material.

Supplementary file1 (186 KB)

## Abbreviations

APR: Acute phase response
AUC: Area under the curve
CDC: Clavien-Dindo classification of postoperative complications
CRP: C-reactive protein
CT: Computed tomography
HCC: Hepatocellular cancer
ICU: Intensive care unit
IL-6: Interleukin-6
IL-8: Interleukin-8
INR: International normalized ratio
NLR: Neutrophil-lymphocyte ratio
ROC: Receiver operating characteristic
TLR: Thrombocyte-lymphocyte ratio
