# Supplementary material for: Interleukin-8 is superior to CRP for the prediction of severe complications in a prospective cohort of patients undergoing major liver resection
Source: Langenbecks Arch Surg. 2023 Sep 25;408(1):377. doi: 10.1007/s00423-023-03041-w (PMC10519863; doi:10.1007/s00423-023-03041-w)
Supplement: Supplementary file 1 — Supplementary file1 (186 KB) [file 423_2023_3041_MOESM1_ESM.docx]

**Supplementary Figure 1:** Occurrence of complications by severity (Clavien Dindo grade) and timepoint.

**Supplementary Table 1**: Resection extent in patients undergoing major liver resection. (PVE indicates portal vein embolization; ALPPS indicates **A**ssociating **L**iver **P**artition and **P**ortal Vein Ligation for **S**taged Hepatectomy; BDA indicates bilidigestive anastomosis)

| **Extent of major liver resections (n=45)** | |
| --- | --- |
| **Extended left hemihepatectomy** | 5 (11%) |
| **Extended right hemihepatecto** | 10 (22%) |
| **PVE** | 14 (31%) |
| **ALPPS** | 4 (9%) |
| **BDA** | 12 (27%) |
| **Major resection with additional  - wedge  - segment 1** | 6 (13%)  8 (18%) |
| **Diaphragm resection** | 3 (7%) |
| **Partial cava vein resection** | 4 (9%) |
| **Small bowel resection** | 2 (4%) |
